# Social Pathways for Ebola Virus Disease in Rural Sierra Leone, and Some Implications for Containment

**DOI:** 10.1371/journal.pntd.0003567

**Published:** 2015-04-17

**Authors:** Paul Richards, Joseph Amara, Mariane C. Ferme, Prince Kamara, Esther Mokuwa, Amara Idara Sheriff, Roland Suluku, Maarten Voors

**Affiliations:** 1 School of Environmental Sciences, Njala University, Njala University Campus, Njala, Sierra Leone; 2 Anthropology Department, University of California, Berkeley, Berkeley, California, United States of America; 3 Development Economics Group, Wageningen University, Wageningen, The Netherlands; 4 Department of Land Economy, University of Cambridge, Cambridge, United Kingdom; Centers for Disease Control and Prevention, UNITED STATES

## Abstract

The current outbreak of Ebola Virus Disease in Upper West Africa is the largest ever recorded. Molecular evidence suggests spread has been almost exclusively through human-to-human contact. Social factors are thus clearly important to understand the epidemic and ways in which it might be stopped, but these factors have so far been little analyzed. The present paper focuses on Sierra Leone, and provides cross sectional data on the least understood part of the epidemic—the largely undocumented spread of Ebola in rural areas. Various forms of social networking in rural communities and their relevance for understanding pathways of transmission are described. Particular attention is paid to the relationship between marriage, funerals and land tenure. Funerals are known to be a high-risk factor for infection. It is suggested that more than a shift in awareness of risks will be needed to change local patterns of behavior, especially in regard to funerals, since these are central to the consolidation of community ties. A concluding discussion relates the information presented to plans for halting the disease. Local consultation and access are seen as major challenges to be addressed.

## Author Summary

Ebola virus disease is a disease of social intimacy. The main infection pathways are through nursing of the sick and through preparation of corpses for burial. In African rural communities these are activities mainly undertaken by close family members. Infection thus spreads to those most intimate with the patient. In effect, Ebola virus disease exacts a high price for family loyalty. It also causes a potential breach between family members and the authorities responsible for imposing infection control. Understanding the social pathways through which infection spreads, and the conflict of human values invoked by infection control are thus key topics in managing the current Ebola epidemic in Upper West Africa. The paper offers a picture, based on case study data from central Sierra Leone, of some of the social factors to be taken into account in efforts to control the epidemic.

## Introduction

### Beyond zoonosis

The present outbreak of Ebola Virus Disease (EVD) in Upper West Africa is the worst ever recorded. As of late December 2014 there were 6808 confirmed EVD cases [1] and there are no clear signs of the disease coming under control. The international community is alarmed, and resources are being rushed to the region to try and stem further spread. The epidemic is an outbreak of the Zaire strain of the virus [2], previously associated with death rates of up to 90 per cent. Death rates in the Upper West African outbreak average 70 percent [3].

The epidemic has been traced to a single index case—the infection of a 2 year-old boy in the village of Meliandou, in the Republic of Guinea [4, 5]. Previous outbreaks of the disease have occurred in remote forest edge communities, e.g. in the Democratic Republic of Congo and Gabon, and have been associated with hunting and eating of bush meat, though human to human transmission also occurred, especially via hospitals. The bush meat scenario is thought to explain the index case, but thereafter appears not to be appropriate for the present epidemic. Human-to-human transmission appears to be the main if not sole source of infection in Liberia, Guinea and Sierra Leone.

**Fig 1:**
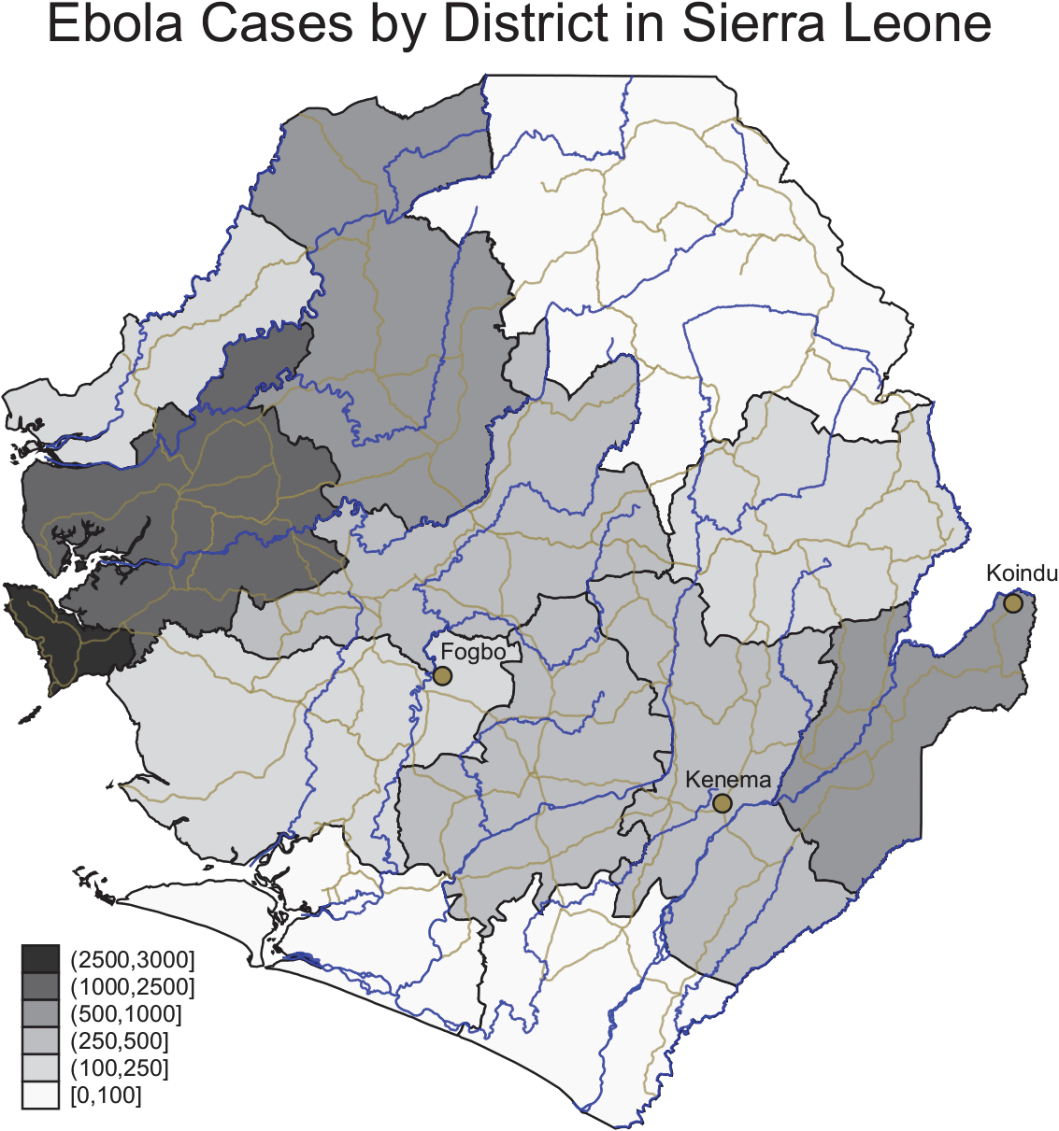
Confirmed Ebola cases by district. Ministry of Health and Sanitation, Situation Report 17 December 2014 (S1 Dataset and S2 Dataset). Figure plots all confirmed Ebola cases by district as of 17 December 2014, and main rivers and roads.

In this paper we offer some data and observations relating to the Sierra Leone epidemic (Fig 1). If human-to-human contact is the main mode of transmission attention needs to be paid to underlying social factors. The paper is divided into three sections. A case-study based scenario for the spread of EVD in Sierra Leone is described (based on interviews and direct observations). We propose that greater attention should be paid to rural buffers for the disease. We then identify and explain the role of processes related to marriage, land and burials significant for spread of the disease. A concluding discussion considers what assistance might be necessary if rural communities are to reduce transmission rates.

### A case of EVD transmission

**Fig 2:**
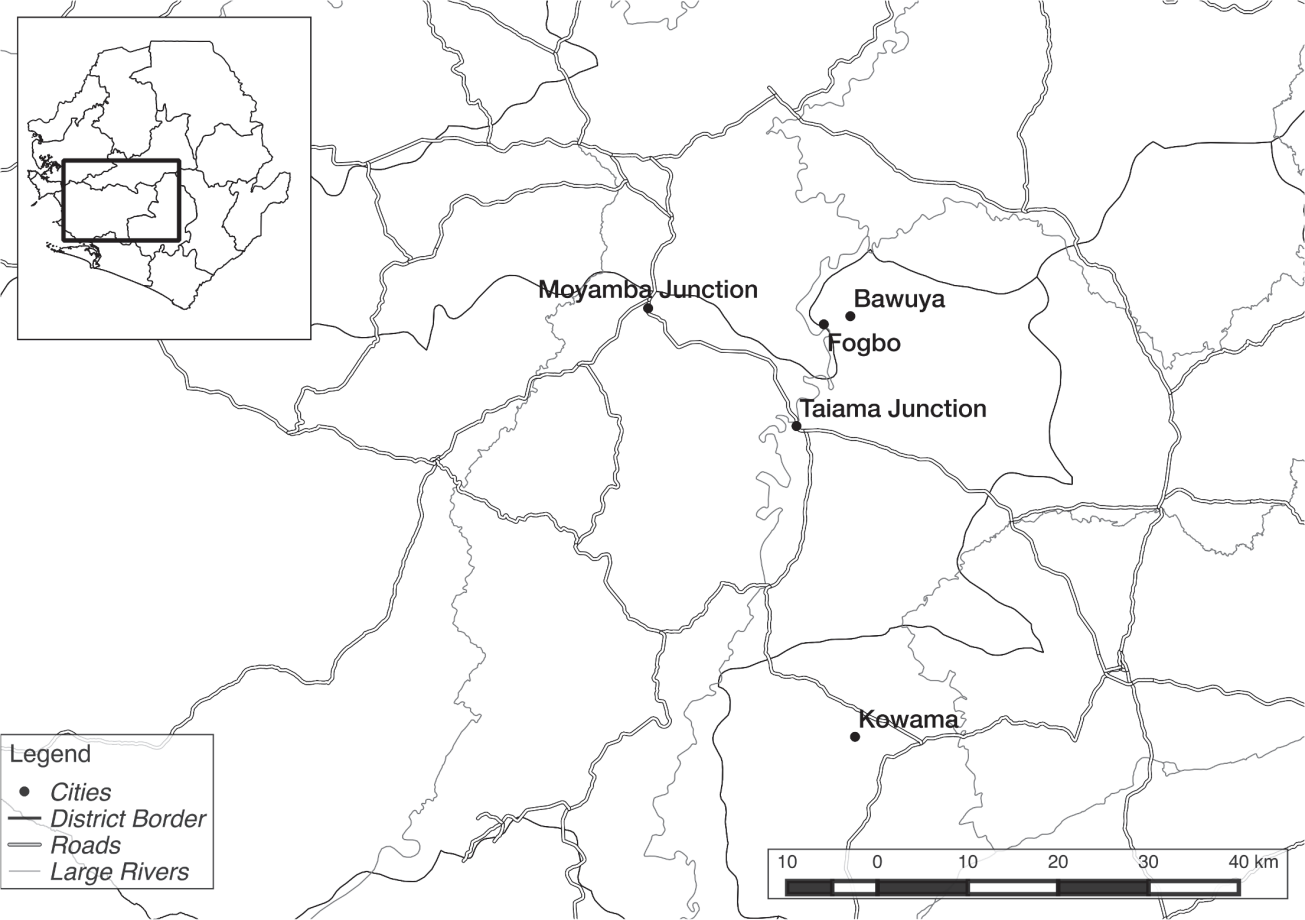
Case study villages. Figure plots case study villages, and main rivers and roads. S1 Dataset.

Fogbo is a Kpa-Mende settlement located on the Taia river about 12 km. north of Taiama, the headquarters town of Kori chiefdom, in Moyamba District (Fig 2). Reachable only by track, the village has a population of about 500 people, larger than average for the region. Reports of Ebola in Fogbo filtered into Taiama in early August. The Community Health Officer visited the village and took a blood sample from a man suspected of having the disease.

The health worker also ascertained that the case was connected to an outbreak in Daru (Kailahun District). Ebola had reached Daru when a wife of the Paramount chief (see Box 1) visited her sick sister, the wife of the Paramount Chief of Kissi Teng, the chiefdom including Koindu market on the Guinea border. A boy infected in the town of Daru came to Kenema, to visit his father. During the visit the young man began to develop symptoms, was taken to hospital, tested positive, and died.

**Box 1. Governance in Sierra Leone**

Non state governance in Sierra Leone consists of a nesting system of chiefs, with speakers and councils of elders, at the village, section, and chiefdom levels. Several villages make up a chiefdom section, and chiefdoms are composed of several sections. The Paramount Chief rules over the chiefdom, and is the highest traditional authority and lowest level reached by the national administration, but a legacy of weak state presence at the rural level has led to considerable autonomy in this office. Chiefdoms are grouped in Districts, and these are grouped in four Provinces. Superimposed on this administrative structure is that of national electoral politics, with demographically-based constituencies (sometimes made up of several chiefdoms) electing their representatives to parliament. Twelve seats in parliament are reserved for Paramount Chiefs, one per District.

The father had also become infected. Apparently not wanting to be hospitalized, he left Kenema by night, evading the curfew, and travelled to his home town Fogbo, where he was cared for by his sister, a Sowei (an elder of the women's secret society—known for her medical knowledge). The town's people and the man's sister did not know he had Ebola.

A few days later the Sowei also became sick. The Community Health Officer was again informed and he took a blood sample, but the Sowei died before the result was available. The villagers concluded, without waiting for the result, that it was Ebola. The town chief called the health officials to come and take charge of the body, but they were unable to attend, and later instructed the people to bury the dead Sowei, but not to wash the corpse.

Prominent women in the community insisted a Sowei respected by her society should be given a fitting burial, so they washed and buried the body. Corpse washing is an important part of local rituals for the dead.

Thereafter, the wife of the town chief was stricken with EVD and died. Since then 16 women and one man have died, all apparently of EVD. By early-September it was reported that somebody in the village was dying every day, and there was nobody to bury the corpses. Local officials sent a message that if the villagers buried the dead without the consent of the government the people would be fined or imprisoned. The Fogbo people waited for the burial team to come. The team had still to reach the village three weeks later.

By this time many people had left the village and gone to live on their rice farms. These farms, often several kilometers from the village, are equipped with simple shelters against the rain, where meals are prepared. More distant farms have special sleeping platforms. Retreating to the farm for days at a time in August-September is normal, since this helps protect the ripening crop from bird and rodent damage, and deters human thieves.

Meanwhile, attendance at the woman's funeral had spread the virus to neighboring villages—Kowama and Bauya, where four people died, and six more were evacuated to an Ebola treatment facility in Kenema. Some of those infected in Kowama sought help from a retired pharmacist in the busy main-road trading center at Moyamba Junction, where the national "lockdown" called in September 2014 revealed both cases and bodies. One schoolteacher in Moyamba Junction died of Ebola at his home in Mile 91, a larger trading location 16 km along the main road to Freetown.

As of 20th September six people in Moyamba Junction had died and others were sick. The case figured on the radio during the government's lockdown period (19–21st September) intended to facilitate tracing of hidden cases of Ebola. It was then reported many people had abandoned Moyamba Junction, perhaps fearing to be quarantined.

The Fogbo case seems typical for the Sierra Leone epidemic, where the disease has moved at times in large jumps along the main road system, passing from town to town, but at other times diverts into the interior to infect isolated villages, where it is little noticed, reported or acted upon, only then to burst out again in a larger town or market center. This pendulum swing between roadside locations and buffer villages in the interior needs to be stopped. Developing effective strategy for this will require close attention to the social factors that allow, or encourage, the virus to spread in more isolated villages.

### Social factors involved in rural Ebola outbreaks

The Fogbo case introduces a number of important social factors in Ebola transmission—notably, the role of the family, marriage, funerals, migration and markets. In this section we focus on each of these factors in turn, and offer some specific data about these variables. The aim is to draw attention to issues to be considered if Ebola control is to be achieved.

We rely on data collected over the past four years during multiple rounds of detailed rural survey work intended to assess levels of rural institutional change in the post-civil war period. Four sources are used: (i) a study of household structures and food security in three isolated communities in northern Moyamba District adjacent to Fogbo, undertaken in May-June 2014, (ii) a national random sample of 2200 rural households in 117 villages in 47 chiefdoms undertaken in 2014 (S3 Dataset), (iii) a survey of 91 villages in 7 chiefdoms around the Gola Rainforest National Park in Kenema, Kailahun and Pujehun districts undertaken in 2013 (S4 Dataset), and (iv) a survey of 187 village communities and 2460 households undertaken in 2010 in the same region (S5 Dataset).

#### Family

Sierra Leonean farming villages are impoverished but self-reliant. This self-reliance was always been a central feature of rural life and was boosted by the civil war in the 1990s (ended in 2002), during which the state withdrew even further from the countryside. In our national survey (S3 Dataset) we included a module asking heads of rural households to assess their degree of trust and reliance in various kinds of institutions.

**Fig 3:**
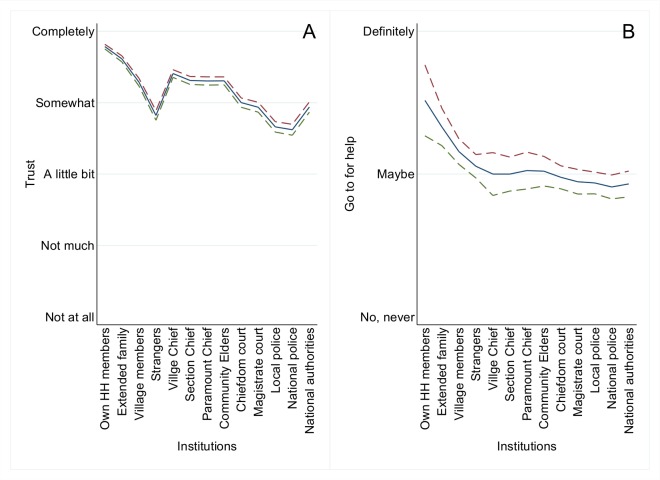
A,B: Trust and seeking help by type of institution. ABC Household Survey (S3 Dataset), 2200 respondents in 117. The graph plots mean response and 95% confidence interval upper and lower bound. Panel A asks respondents “How much do you trust [institution]? “, responses are on a five point scale ranging from “Not at all” to “Completely”. For Panel B, respondents are asked “If you were in trouble, would you go to these people for help?”, responses are on a three point scale ranging “No never” to “Definitely”.

In Fig 3A, we present data on how respondents rate their trust in various institutions. The data show that while overall trust is high, inter-personal relations are perceived as more trustworthy than those with institutions beyond the local level. Trust is highest in household members and extended family. Conversely (at the local level) there is a noticeable distrust of "strangers" (persons born outside the local community). Thereafter there is a general decline in trust as the scale of the institution expands beyond the local level. Trust in central government, however, is above the trend.

When we asked about assistance (to whom would the person interviewed turn for help, Fig 3B) the pattern was somewhat different. As the scale of institution increases outwards there is a steep decline in confidence in help coming from beyond the family. Central government is no longer above trend. In effect, rural people, across the country, seem strongly to expect to find assistance mainly their own immediate family group. In a crisis it is sensible to head for home.

This finding seems relevant to understanding the Ebola epidemic. Respect for and trust in authority is quite high. It is highest for local authorities but also reasonably high for central government. Most communication around Ebola has been from chiefs, district council representatives, parliamentarians and ministers. There is some indication that these messages have been effective in changing awareness at the local level. In an August 2014 survey conducted in seven districts, most people said they now believed the Ebola epidemic is real, and that Ebola cases should be isolated in hospital [6].

But there is also a widespread impression that little can be done by health professionals to alleviate the disease. The different curves in our data on trust and reliance capture the tension between what people say, and what they actually do. They say they would seek assistance from a hospital, but in practice they hesitate to go. Of course, poverty and distance and poor transportation are an important factor in explaining why people fail to seek health care at state institutions. At the same time, in the final instance it is the family, the most trusted source of reliable assistance, that will help them cope. As is apparent in the Fogbo case, the move by an Ebola sufferer back to an isolated rural home then buffers the spread of the disease.

At present, attention is focused on local opinion leaders (Muslim imams and Christian pastors) to help change attitudes, to reduce the threat posed by burials, and to bring Ebola cases into isolation facilities. These figures are, indeed, widely respected. But there is also skepticism among villagers about whether these opinion leaders can provide practical help. If an Ebola victim in a rural village is to be brought to an isolation facility it will be the family that makes the arrangements and does the work. Our data suggest that assistance should also to be targeted on rural families.

#### Marriage and funerals

This then implies a need to understand Ebola risks from the perspective of family, and its notions of inescapable social obligations. This includes obligations to both the dead as well as the living. In the first instance, this double obligation arises from the system of access to land, the basic resource for survival in a peasant agrarian economy.

Sierra Leonean ethnic groups are predominantly patrilineal. Villages are formed from several patrilineal groups (typically, perhaps, 4–10 per village). Each group maintains a shared right to land for farming, and generally occupies a specific quarter within the settlement. There may be some ancestral graves or a family shrine within the quarter.

In Mende-speaking communities such as Fogbo, not all residents of a quarter, however, are members of the patrilineage [7]. Recent surveys (May-June 2014, details above) for three villages in northern Moyamba District, in the general neighborhood of Fogbo show that only about 40 percent of residents of each quarter belonged to the patrilineage. This is because lineage exogamy is the norm in rural Sierra Leone, and wives resident in a family quarter will come from outside the husband's lineage.

Some wives come from other lineages in the village, while others come from other villages. Most of these outside marriages are local, but some link distant communities. This is especially likely with ruling families (lineages with a recognized right to compete for chieftaincy). Historically, ruling families consolidated power by making advantageous unions [8, 9] This practice continues today, and is relevant to the story of the spread of Ebola.

In-marrying women from other villages will be termed "stranger" (*hota*, in the Mende language spoken in Fogbo). While married such a woman can access land for farming from her husband's lineage, and if she becomes a widow she will be strongly encouraged to take another husband from the same lineage (through the institution of levirate, see Box 2). But if she rejects this option, or the marriage fails and (especially) if there are no children, a *hota* wife may be required to return to the village where her brothers maintain their own land rights.

**Box 2. Caretaker Husbands**

In Mende villages, no woman lacks a husband. If she is a widow she will expect to have a caretaker husband. This husband is not necessarily resident with the woman or in any kind of sexual relationship. He serves as the socially recognized figure needed for certain kinds of ritual transactions, especially those surrounding death and burial. The caretaker husband is less active when the woman is alive, but when she dies, he is the one the town chief and elders of the town will look up to for the funeral ceremony. The caretaker husband will have to contact the relatives of the dead woman before any action is taken. If the relatives have to come, they will be in the care of the caretaker husband, and it is this husband who will have to convey the corpse of the deceased to her home. So the chances of a caretaker husband contracting Ebola are likely to be high. In the case of incomplete marriage, tradition decrees that the close male relatives of the husband (those who would normally negotiate and convey the marriage payments) take charge. In case they are no longer alive the elders of the husband's family will assume responsibility for the funeral ceremony, because they are the ones that begged for the life of the daughter from the relatives to give to their son. If women are at high risk from nursing patients with EVD and washing widows men have high chances of contracting the disease in matters regarding the inter-village transfer of corpses.

Source: RS, field-notes August 2014, edited PR

This concern to avoid merging of lineage land rights through marriage is especially strongly maintained where marriage takes place between ethnic groups, in places such as Fogbo, which stands on the border between the Mende and Temne speaking areas. Funerals are important not just to mark the passing of the deceased but also as part of the process of "unmaking" a marriage at death, so that families can publicly reassert their land rights and decide whether a union is to be continued (to be "remade" through levirate marriage) or is to be finally dissolved, with the woman returning to her own natal community.

To carry out a funeral properly a number of things need to happen. The corpse has to be washed, and this is thought to be an especial point of danger for Ebola transmission. Men wash men's bodies and women wash women's bodies. The women will include the deceased woman's sisters, and this risks spreading the Ebola virus to other lineages and (where the woman was *hota*) to other villages.

Where a man died, the wife then has to have her head shaved and be covered with mud formed from the washing of the husband's corpse [10: pp 94–97]. This is part of a ritual that frees her from the attentions of the dead husband's jealous spirit, and prepares her to be remarried to one of his brothers, or to return to her own family. This also seems a likely high risk factor for Ebola transmission.

Where the deceased is an in-married *hota* wife it may be necessary for the corpse to be returned to her family living in another village. This is a possibility where the marriage payments are not yet complete. A relationship begins upon agreement that certain gifts and services will be provided for the parents and family of the woman by the husband in recognition of that lineage's gift of a bride. Certain offerings are made to initiate the relationship, but the marriage is incomplete until everything promised is fulfilled. A prospective son-in-law offers labour and material help to his partner's parents for many years before he can say the marriage is complete.

In the case of incomplete marriage, the male partner will first travel to the dead wife's village to make a settlement of outstanding marriage payments before being permitted by her patrilineage to bury the corpse. If he is unwilling or unable to pay, he loses the right to control the burial arrangements, and his wife's family are also within their rights to claim the children from the marriage. Only when settlement is reached can the man presume to bury his wife in his home village. But if he is unwilling, or cannot, settle the debt the corpse will have to be returned to her own patrilineal kin for burial. The task of transporting the body will fall to men, probably using a hammock, and is obviously a highly risky practice where Ebola was a cause of death.

How frequent is the problem? Incomplete marriage is far from uncommon. In the study of three villages mentioned above data were collected on 79 current marriage partnerships (see [11]). In 62% of cases the female partner was a stranger (ie. came from another village). These stranger marriages were in most cases incomplete, the marriage payments were only fully finalised in 16% of cases (The figure was even lower for citizen marriages [*tali*]—i.e. between lineages from the same settlement.)

Without funerals, orderly access to farm land for staple rice production—a key survival requirement for rural families—is seen to be at risk. Land is currently an especially sensitive issue due to numerous sales and leases to mining and agri-business concerns. This helps explain the tenacity with which villagers defend funeral practices, despite official injunctions on unauthorized funerals, imposed as an Ebola infection control measure. The Fogbo people waited some time for an official burial team, but when trained workers failed to appear they did what they felt necessary to protect their family concerns. They washed and buried the corpse as the dignity of the deceased woman demanded. Thereby lineage rights, land tenure, and inter-family and inter-village relations were maintained, even though Ebola spread.

#### Migration

The Fogbo case study brings out the importance of long-distance social networking resulting from migration, and the importance of trading patterns and market centers. Here we assess these risk multipliers in terms of data relating to typical patterns of village inter-dependency, based on motivations for labor, education and marriage migration, and distance to markets.

Villages are not independent units scattered across the landscape, nor is it the case that villages all fall into a system based on a clear functional hierarchy of administrative or market centers. The landscape of rural Sierra Leone bears the marks of a long, complex, and often violent, history. This heritage is apparent in physical markers, but also in rather complex patterns of local social interaction.

Old inter-family animosities (some revived by the recent civil war) still disrupt local interaction. Neighboring settlements may, for instance, be old enemies, and might not readily cooperate over sharing facilities. This has particular relevance to the siting of local Ebola holding centers. Equally, unexpected patterns of cooperation link more distant communities, in the case of trading relations and marriage alliances. These patterns, hard to anticipate without detailed local knowledge, influence the spread of Ebola, blocking it in some areas, but in other areas opening up unanticipated channels of infection. Cross-border family-based and market networking among Kissi-speaking settlements divided by colonial boundaries into three separate states (Guinea, Liberia, and Sierra Leone), has been a major factor behind the rapid build-up of the disease in its epicenter.

**Fig 4:**
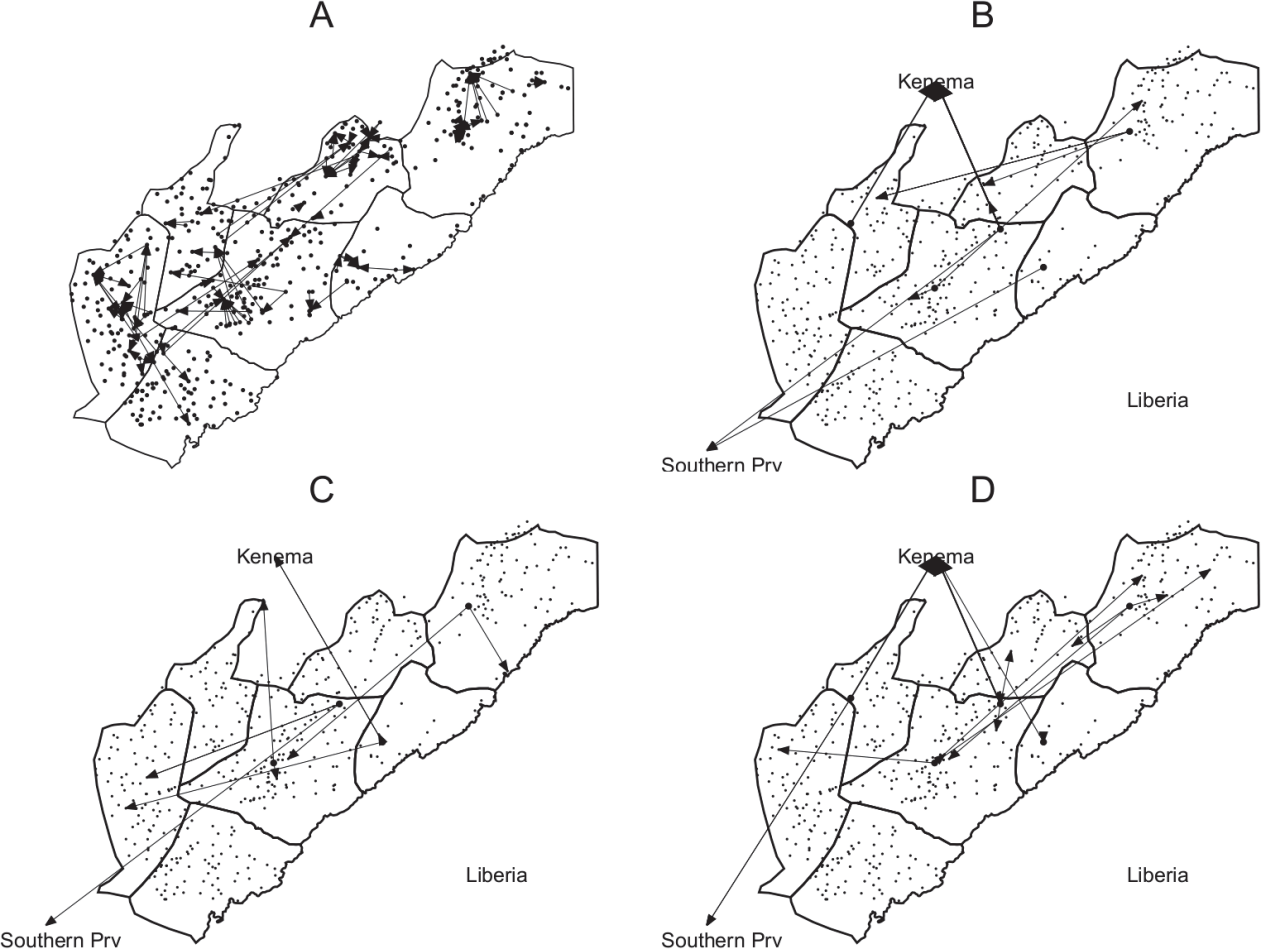
a-d: Village dependencies, migration for marriage, work and education. Figure (a) plots village social, economic and political dependencies. Source: Community Survey (S4 Dataset) in 91 villages in Eastern Sierra Leone in Malema, Makpele, Nomo, Gaura, Tunkia, Koya and Barri Chiefdoms. Map plots the village dependencies as indicated in a community focus group meeting with arrows going to the location the village depends on socially, economically and politically. Figure (b)-(d) use Household Survey (S5 Dataset) under 2460 respondents in 187 villages. Figure plots the migration patterns (origin and destination) for six randomly selected villages for household members who left the household for marriage, work or education during 2000–2010. In total we recorded data on 4208 migration decisions. Of these, 20% are for marriage, 36% are for attending school and 17% to work elsewhere. Work migration is for trading, mining, labourer for cash crop production and urbanisation. Location of Kenema is approximate.

Our data are intended to convey a picture of some of this local complexity, relevant to understanding of rural Ebola transmission risks. Below, in Fig 4A, we picture some typical village dependencies in seven chiefdoms bordering the Gola forest. All seven chiefdoms look to Kenema as their regional administrative and market center. The map is based on asking focus groups of village elders in 91 villages what other villages they depended on, in terms of political, social and economic relations. Arrows point to the village depended upon.

There are some obvious patterns, such as those in Tunkia chiefdom, where there are numerous small villages dependent on one town Golahun, the chiefdom headquarters. This is an area where settlers moved up to the forest edge and established satellites in the past half-century. But rather more unexpected is the number of villages linked in quite long lateral ties of dependency. These in particular follow the grain of a major pre-colonial trading route along the western side of the Gola forest to the coast in Pujehun district. Some of the connections cross chiefdom boundaries, and reflect ties based on the politics of elite marriage alliance.

A second set of maps, Fig 4B–4D, focuses on the movements of young people into and out of villages for marriage, schooling and work. Apart from marriage, education is the single biggest cause of inter-village or village-to-town movements in rural Sierra Leone, except for the special case of movements to the mining areas (which we do not address). Here we have plotted data for 6 randomly chosen villages from the 187 villages included in the 2010 survey (S5 Dataset), related the destinations of people who left the household.

Again, the same mixed pattern emerges. As expected, Kenema shows up as a destination for marriage, work and school, but once again there are a good number of unanticipated lateral linkages. These lateral connections are on a quite large scale, crisscrossing the entire forest-edge region.

If marriages offer a clue to the pattern of movements that funerals will one day generate, then mourners are likely to come from across the region. Thus we should not be surprised to find that a funeral of the wife of a chief in a Kissi chiefdom on the Guinea border generated an Ebola outbreak in a Mende chiefdom (Daru), where the wife of the chief, and sister to the chief's wife in Kissi, also died from the disease. Daru was the ultimate source of the Fogbo outbreak.

In other cases, movements to and from school, or migrant workers returning from distant locations, may also have spread the disease. One such case is the outbreak in Sahn, Malen chiefdom, Pujehun District, triggered by a student from Kailahun District reportedly visiting his uncle during the school holiday period [12].

#### Markets

Market data show similar levels of complexity, and once again the risks of Ebola transmission can only be fully understood with some grasp of historical connectivity.

The index case for EVD in the Upper Guinean forest region is a small child who died of the disease in the village of Meliandou in early December 2013. The disease spread to Gueckedou, a city of 200,000, 8 km. distant from Meliandou, and then to neighboring cities of Macenta and Kissidougou, and along international roads crossing to Liberia (Lofa County) and Sierra Leone (Kailahun District).

The area is sometimes seen as remote and impoverished. This needs some qualification. Historically, the area around Gueckedou was at an important junction for intra-West African trade, carried between the coast and interior savannas along two major trade routes on the eastern and western sides of the Gola forest [13]. Even today, traders and smugglers carry kola nuts and gold from the margins of the Gola forest and enclaves within it [14]. In the late 19th century the area immediately north of the forest was a veritable entrepot for international trade [15].

Thomas Alldridge, a British travelling commissioner, who visited the region in 1893, on the eve of colonial conquest, introduced his account of what he termed an "ordinary native market" in the area with the remark that "*I think I shall be able to show that these up-country people are not at all in the wretched condition often pictured by the European imagination*" [16].

Alldridge lists the diversity of products available for sale, bought and sold in the local currency, "Kissi pennies". Elements of this regional trade survived colonial conquest, and in 1932 the British in Sierra Leone allowed a large international market to open at Koindu, very close to the Guinea border. Koindu market was closed during the 1990s because of war, but has revived since 2008.

Koindu (only 30 km from Meliandou) had a large number of cases of EVD in May-June 2014, and a further surge of cases (perhaps crossing from Liberia) was reported late September. Koindu's role as a major booster of the epidemic might have been better anticipated given its history of involvement in intense cross-border commerce.

#### Reaching help

International efforts are focused on establishing a more widespread network of secure, well-run isolation units. These units need to be attractive to Ebola sufferers who have hitherto shunned voluntary hospitalization. A problem of accessibility needs to be addressed. Victims in the interior villages will often need to overcome a major distance barrier in reaching such centers.

In a recent national random sample of 117 villages in 47 (out of 149) chiefdoms we ascertained how close are typical classes of village to their chiefdom headquarters. The sample compares well-connected villages where agri-business centers have been located with typical less well-connected villages without agri-business centers located within the same chiefdom section (off-site villages). The same question was also asked in the survey of 187 Gola forest edge (S5 Dataset) villages. These are among some of the most inaccessible places in the country.

**Fig 5:**
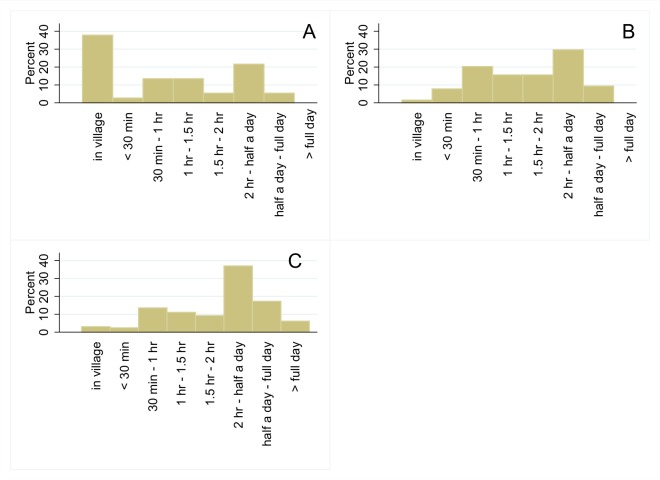
a-c: Distance to Chiefdom headquarters town. Source: Figure plots the distance frequencies to the Chiefdom headquarter towns for villages with an Agri-Business Center (Panel A (S3 Dataset), villages without an Agri-Business center (Panel B, (S3 Dataset)) and villages around the GRNP (Panel C, Household Survey (S5 Dataset)).

The data are tabulated in Fig 5A–5C, and show the time distance separating the village and chiefdom HQ in the three types of settlement. Even in the case of the best-connected group (agri-business center villages), 10 per cent of settlements were more than two hours to a full day from their chiefdom headquarters. For the Gola forest villages the percentage rises to about one quarter. These distances would be insurmountable by an Ebola victim seeking to walk to voluntary hospitalization sites. A wealthy family might pay for a hammock. Others would be unable to afford to get their patient even to a "local" triage facility. Perhaps only helicopters could solve the problem of timely evacuation from localities that are not reached by motorable roads.

## Discussion

In the Fogbo case, discussed above, a carrier exposed to the disease in an urban location traveled towards a rural village, where family help and local remedies were sought. Distance frustrated attempts by the authorities to impose isolation and safe burial. This set up a new incubation focus for the disease, which spread locally without attracting further attention. Traders seeking rice and other food commodities then interacted with this local rural focus of the disease and drew the virus back towards market centers and the main roads, triggering further expansion of the epidemic.

What does this pendulum swing imply for attempts to control the disease? It is generally agreed that rapid identification, removal and isolation of cases for treatment is essential. This is made more complicated if the disease periodically dives into the backcountry. Drawing it out will require some ingenuity.

The scale of the epidemic makes the active following of cases into the interior practically impossible. The alternative is for the unwell to seek out testing and treatment voluntarily, and in sufficient numbers to bring the reproduction rate of the disease below 1.0.

For this to work, our analysis above suggests there are three key tasks. First, accurate information has to be conveyed in interior settlements about the true causes of the disease and infection pathways. A survey in summer 2014 of attitudes in seven districts in Sierra Leone suggested that whereas many people accepted the reality of Ebola, more than 80 percent still thought it was caused by eating bush meat [6]. The role of funerals, body washing, social networking and market exchange in spreading the disease also needs to be carefully explained.

Second, the option of seeking hospitalization has to be made truly attractive to the sick. This means that evidence the disease can be survived must be made more widely known. Survival rates in treatment centers also need to be boosted beyond a current estimated 35 percent [17]. These authors suggest ways in which this can be done.

Local triage centers will also need to offer effective rapid testing, guaranteed medical supplies for treating other diseases, and reimbursement of travel costs incurred by the unwell and their family carers. Moving a very sick person out of an isolated village across non-motorable tracks is a major deterrent to referrals. The difficulty, expense, and ordeal is so high that it often seems better to the family not to move the patient and let God decide.

Often a hammock (perhaps three or four times more expensive than a motor bike taxi) is the only realistic transport option. The costs to families, and the risks to helpers, should be fully assessed and built into the way any triage centers work, if they are to attract cases from interior villages.

Third, attention has to be focused on how family groups in the villages can protect themselves from the disease when all other options are foreclosed. Families will not readily abandon their relatives in extreme crisis, so they need information on how to minimize the risks associated with tending sick patients. This is now addressed in a poster [18] offering guidance on "what to do while you wait" (for arrival of an Ebola ambulance). In some cases, this wait can be indefinite [19]. The poster describes the need for one member of the family only to be designated as a nurse, and for all other members to provide support and encouragement, but only from a distance. Early use of oral rehydration salts [ORS] is recommended, coupled with advice on supplying this without the nurse directly handling the drinking cup. This now needs to be followed by some attention to practical items often lacking in village conditions (e.g. sufficient supplies of soap, disinfectant, protective clothing, rubber gloves, buckets and ORS).

Some guidance is also now available in the form of a protocol from the World Health Organization [20] covering safe and respectful burial in village conditions. Villagers are very clear (see S1 Text) that mandatory Ebola burials, carried out by one or other of the 90 or so national cadre of trained Ebola burial teams have caused considerable difficulty, due to haste and disrespectful treatment of bodies. At times this has amounted to little more than sanitary disposal. The protocol is a great improvement, since it now specifies that a pastor or imam be present in all Ebola funerals, and the involvement, at a distance, of family witnesses. But as yet the protocol does not allow enough local input to accommodate the sociological concerns about debt and inheritance mentioned above. It is important for these issues to be faced by those in charge of burial teams, since it will open the door to better local cooperation.

Flexibility over burial ritual was already apparent even before the advent of Ebola. Where it was impracticable to bring a body home for burial the family pragmatically accepted that the funeral had to be organized in the place where death occurred. Further flexibility will be encouraged if villagers participate in focus sessions where they have an active hand in agreeing on the safest compromises. Corpse washing should be discouraged, but if it cannot be avoided then it should be done only with biohazard protection. Families should be encouraged to meet to agree to postpone outstanding final marriage settlements for the duration of the epidemic.

In coming together to debate these issues villagers might also be encouraged to form village health clubs, to develop informal community "bye-laws" (club rules) to regulate against the most dangerous practices. In the case of Ebola, these rules might specify acceptable funeral practices, and when, why and how to quarantine patients if no other options present. In some Ebola epidemics villagers have resorted to building temporary shelters adjacent to settlements to care for suspected Ebola victims. Similar developments might be encouraged through village health clubs in Sierra Leone. Rules governing care provision while waiting for assistance need to be debated and agreed, e.g. to limit the number of carers to one person per family.

The Ebola epidemic in Upper West Africa is the largest ever seen, and Sierra Leone is now the most seriously affected country. The international community perceives the epidemic as a threat to global security, and an abundance of help has now been provided to all three countries. Experts agree that with logistics in place containment should be a straightforward task. One thing that could blow this assessment off course is the persistence or revival of rural buffers of the disease. In Sierra Leone these are not found in forest-edge communities associated with zoonotic transmission but in the much more numerous farming villages that incubated the rebel war of the 1990s, due to inaccessibility and poor communications. An effective approach to control of Ebola Virus Disease requires detailed knowledge of these interior rural landscapes and how they function, including the key part played by rural-urban extended family networks. In turn, this knowledge should feed effective planning to extinguish the numerous further localized outbreaks that can be expected as a result of a now rampant urban epidemic feeding back upon far-flung rural locales as a result of dense rural-urban family and economic networking.

## Supporting Information

S1 Text Post script. (DOCX)

S1 Checklist STROBE Checklist for cross-sectional studies. (DOC)

S1 Dataset Shape files of national, District and Chiefdom boundaries, important roads and rivers. (ZIP)

S2 Dataset Confirmed EVD cases by district, source Sierra Leone Ministry of Health http://health.gov.sl/
(ZIP)

S3 Dataset National random sample of 2200 rural households in 117 villages in 47 chiefdoms undertaken in 2014. (ZIP)

S4 Dataset Survey of 91 villages in 7 chiefdoms around the Gola Rainforest National Park in Kenema, Kailahun and Pujehun districts undertaken in 2013. (ZIP)

S5 Dataset Survey of 187 village communities and 2460 households undertaken in 2010 Kenema, Kailahun and Pujehun districts. (ZIP)
