# Supplementary material for: Social Pathways for Ebola Virus Disease in Rural Sierra Leone, and Some Implications for Containment
Source: PLoS Negl Trop Dis. 2015 Apr 17;9(4):e0003567. doi: 10.1371/journal.pntd.0003567 (PMC4401769; doi:10.1371/journal.pntd.0003567)
Supplement: S1 Text — (DOCX) [file pntd.0003567.s001.docx]

**Postscript, December 20th 2014**

Ebola virus disease has four phases: a few days from infection to expression of first symptoms, a so-called "dry phase" of a few days where the symptoms largely resemble those of other diseases such as malaria, and an equally short third "wet" phase often resulting in death, followed by a post-mortem phase where the corpse remains highly infectious until buried.

The phases largely determine the infection risk. During the second phase the patient becomes aware of illness, and begins a search for assistance, but without recognizing Ebola. The few days in question are sufficient for long distances to be traveled. These movements - illustrated in the case study offered above - help explain the non-linear character of the epidemic, here termed a pendulum effect.

A shift in epidemic focus over the last three months of 2014 from eastern borderlands to the capital territory does not mean an end to this pendulum-like oscillation of infection between rural and urban communities. The family remains the main source of reliable support in a crisis, and an urban migrant will wish to head for the family home in the countryside at the first signs something is seriously wrong.

When symptoms show this to be a likely Ebola case the person has in effect often moved beyond the range of the rapidly-built and largely urban-based biosecure Ebola facilities built with the help of the international community, but is now in the "wet" phase and too ill and dangerous to be moved. The waiting time for assistance is long. Advice on "how to care for a patient until help arrives", and "safe burial", remain crucial aspects of Ebola control.

Given the continued likelihood of rural outbreaks, reignited by cases among urban migrants returning to the extended family home, it was decided to undertake a re-study of the Fogbo case described above. How well did villagers now understand the disease risks, and what steps were they taking to prevent reinfection. These data will be the subject of a subsequent paper in preparation, but it seems important briefly to mention a few key findings as an update to the case presented above.

There have been major changes in perceptions of behavior related to the disease. Occult explanations have been dropped in favor of understandings framed in of the risks posed by body fluids. Whether as a result of messages from health teams or through local discovery, houses with Ebola cases have been quarantined, and "one-on-one" caring adopted. The food trade at Moyamba Junction has largely been suspended, and some market women have taken to making and selling charcoal instead. Villagers are now very aware of the risks posed by health-seeking visits by migrant members of extended families. Visitors are prevented from passing the night in the villages. Active transmission of Ebola Virus Disease now appears to have been ended.

Where there is still concern and dissension is over the activities of burial teams. Preparing the body for the afterlife (including the need for an imam and assistant to enter the grave to ensure the body is turned towards the rising sun) remains a strongly felt need. Funeral rituals cannot be abolished, especially where they speak to the power and influence of elders or to the transfer of social identity and property rights from dead to living. No clear procedures have yet been established to put these requirements into temporary abeyance to address the Ebola infection risk. Burial is now safer, and more respectful, but it is not yet adequate to the needs of societal reproduction. Efforts at consultation with and engagement of local groups on this crucial issue must continue, or dangerous evasions will continue to be attempted.
